# Liraglutide Lowers Palmitoleate Levels in Type 2 Diabetes. A *Post Hoc* Analysis of the LIRAFLAME Randomized Placebo-Controlled Trial

**DOI:** 10.3389/fcdhc.2022.856485

**Published:** 2022-03-04

**Authors:** Asger Wretlind, Emilie Hein Zobel, Andressa de Zawadzki, Rasmus Sejersten Ripa, Viktor Rotbain Curovic, Bernt Johan von Scholten, Ismo Matias Mattila, Tine Willum Hansen, Andreas Kjær, Henrik Vestergaard, Peter Rossing, Cristina Legido-Quigley

**Affiliations:** ^1^Steno Diabetes Center Copenhagen, Herlev, Denmark; ^2^Department of Clinical Medicine, University of Copenhagen, Copenhagen, Denmark; ^3^Department of Clinical Physiology, Nuclear Medicine & PET and Cluster for Molecular Imaging Copenhagen University Hospital, Rigshospitalet, Copenhagen, Denmark; ^4^Department of Biomedical Sciences, University of Copenhagen, Copenhagen, Denmark; ^5^Novo Nordisk AS, Bagsvaerd, Denmark; ^6^Bornholms Hospital, Rønne, Denmark; ^7^Institute of Pharmaceutical Science, King’s College London, London, United Kingdom

**Keywords:** liraglutide, GLP-1 RA, palmitoleate, palmitoleic acid, stearoyl-CoA 9-desaturase 1 (SCD1), type 2 diabetes (T2D), monounsaturated fatty acid (MUFA)

## Abstract

**Background:**

Liraglutide is a glucose-lowering medication used to treat type 2 diabetes and obesity. It is a GLP-1 receptor agonist with downstream metabolic changes beyond the incretin system, such as reducing the risk of cardiovascular complications. The understanding of these changes is critical for improving treatment outcomes. Herein, we present a *post hoc* experimental analysis using metabolomic phenotyping to discover molecular mecphanisms in response to liraglutide.

**Method:**

Plasma samples were obtained from The LiraFlame Study (ClinicalTrials.gov identifier: NCT03449654), a randomized double-blinded placebo-controlled clinical trial, including 102 participants with type 2 diabetes randomized to either liraglutide or placebo treatment for 26 weeks. Mass spectrometry-based metabolomics analyses were carried out on samples from baseline and the end of the trial. Metabolites (n=114) were categorized into pathways and linear mixed models were constructed to evaluate the association between changes in metabolites and liraglutide treatment.

**Results:**

We found the free fatty acid palmitoleate was significantly reduced in the liraglutide group compared to placebo (adjusted for multiple testing p-value = 0.04). The activity of stearoyl-CoA desaturase-1 (SCD1), the rate limiting enzyme for converting palmitate into palmitoleate, was found significantly downregulated by liraglutide treatment compared to placebo (p-value = 0.01). These metabolic changes have demonstrated to be linked to insulin sensitivity and cardiovascular health.

## Introduction

An increasing number of people worldwide are diagnosed with type 2 diabetes and are in need of pharmaceutical therapy to manage their blood glucose (1). However, with multiple anti-hyperglycemic therapies available, an improved mechanistic understanding of these drugs is needed for their efficient use (2, 3). Liraglutide is a GLP-1 receptor agonist and a medication utilized for lowering blood glucose in people with diabetes and for inducing weight loss in people with obesity. Liraglutide was designed to mimic endogenous GLP-1 and binds to the GLP-1 receptor triggering insulin secretion (4), yet displaying effects beyond the incretin system. Liraglutide shows additional effects such as weight loss (5–10), reduces the risk of cardiovascular diseases (11–13) and improves the lipid profile (14–17). These mechanisms, while crucial to sustain long-term health, are not well understood. Using lipidomics we reported widespread changes to the circulating lipidome after liraglutide treatment; particularly unsaturated triglycerides, phospholipids and ceramides were reduced by liraglutide (18). Given these observations, we hypothesized that small polar and bioactive lipids could be further involved in liraglutide induced metabolism. To this end, we have also measured polar small molecules using metabolomics to discover novel insights in this complex mechanism (19, 20).

In the present study, we aimed to investigate the metabolic changes that follows liraglutide treatment compared to placebo in people with type 2 diabetes, using mass spectrometer approaches and uncovered palmitoleate and SCD1 metabolism as possible mediator in lipid changes induced by liraglutide.

## Materials and Methods

### Clinical Trial

Plasma samples were acquired from the clinical trial The LiraFlame Study which has previously been described in detail (21) and registered at ClinicalTrials.gov with the identifier: NCT03449654. In brief the trial consisted of 102 participants with type 2 diabetes, age > 50 years and HbA_1C_ ≥ 48 mmol/mol. Participants were randomized to receive daily subcutaneous injection of liraglutide (up to 1.8 mg daily) or placebo treatment for 26 weeks. The maximum dosage of 1.8 mg/day were reached in 70 of the 102 participants as per protocol in an average of 18 days. From the remaining 32 participant 8 were given full dose by the end of the trial, 12 had their dose reduced and 12 discontinued treatment before week 26. An overview can be found in **Supplementary Table 1**. The Primary outcome of change in vascular inflammation assessed by FDG PET/CT was not reached (21). Participants were receiving standard care in addition to the trial. Plasma samples were collected for analysis at baseline and end of treatment. Participants were told to be fasting for 4 hours prior to blood sampling. Five participants dropped out and did not have blood samples taken at the end of the trial and was therefore not included for the statistical analysis.

This study was carried out in concordance with the principles of the Declaration of Helsinki and ethics approval was granted by local ethics committee (H-16044546) and the Danish Medicines Agency (2016110109). Participants provided written informed consent before being included.

### Metabolomics

Metabolites were measured *post hoc* in the blood plasma with an untargeted approach using two-dimensional gas chromatography coupled to a time-of-flight mass spectrometer (GC×GC-TOFMS) from LECO Corp. This technique has been fully explained by Pedersen et al. (20). Data preprocessing, peak matching, alignment and normalization were performed using ChromaTOF software from LECO Corp. and Guineu (22).

A panel of 31 metabolites associated with diabetes and metabolic dysregulation were also measured and quantified using a targeted method based on ultra-high-performance liquid-chromatography linked to a triple-quadrupole mass spectrometer (UHPLC QQQ-MS/MS) from Agilent Technologies as reported by Ahonen et al. (23). Metabolites measured in both methods were compared for technical validation.

### Statistics

#### Pathway Analysis

Metabolites were classified into pathways adapted from Green et al. (24). Pathways with less than 4 metabolites were not included, resulting in 8 investigated pathways. Metabolites within each pathway were z-transformed, the mean was then used to create a combined score for each pathway. Linear mixed models for each pathway was constructed, explaining pathway score as a function of treatment and time, allowing random effects between participants to evaluate which pathways were changed by treatment.

#### Single Metabolite in Selected Pathway

To determine which metabolites were affected by liraglutide treatment compared to placebo we created linear mixed models for each metabolite, explaining metabolite level as a function of treatment and time, allowing random effects between participants. Adjustment for sex, change in BMI, change in HbA_1c_. Treatment dose, use of lipid lowering medication (statins) and thiazolidinedione treatment was also evaluated. Data analysis and visualization were performed with R (25). Linear mixed models were fitted using the lme4 package in R (26) and the models were visualized using ggplots and ggeffects also in R (27, 28). P-values were corrected for multiple testing using FDR correction. All metabolites were log10 transformed prior to analysis. Effect size between the two treatment groups were calculated using the effsize package in R (29).

#### Enzyme Activity

The ratio of plasma fatty acid product and substrate were used as surrogate for enzyme activity. SCD1 activity was calculated as the product-to-precursor ratio between palmitoleate and palmitate (30–33).

#### Metadata Exploration

Correlations of metabolites and clinical measurements were investigated and visualized using corrplot package in R (34) with a cutoff of minimum 30% correlation, this analysis integrated lipids from our previously reported lipidomics data (18). A mediation analysis was carried out on metabolites of interest, testing if their association to liraglutide treatment compared to placebo was mediated by change in BMI, this was done using linear regression models, the effect and significance was estimated by generating 500 sets of bootstrapped data using the mediator package in R (35).

## Results

**Figure 1:**
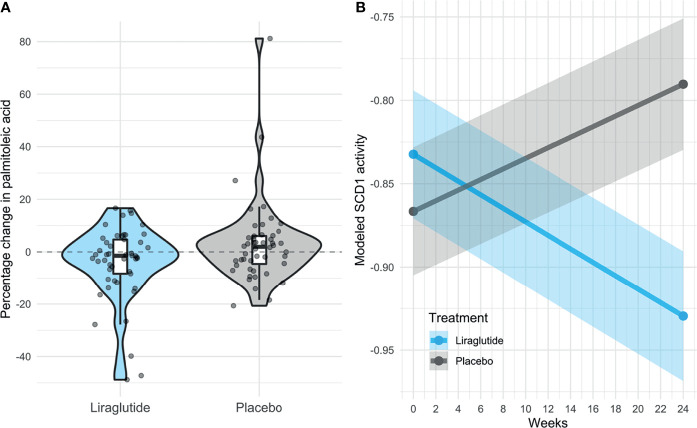
Level of palmitoleate and SCD1 activity. Change in levels of Palmitoleate and SCD1 after treatment with Liraglutide. **(A)** Change in palmitoleate during the trial in percentage, comparing distribution between liraglutide treatment and placebo. **(B)** SCD1 activity with liraglutide treatment and placebo in participant with type 2 diabetes (n =97). The SCD1 activity is fitted to observation at baseline and after 24 weeks of therapy using a linear mixed model that allows for random effects between individuals. SCD1 activity ~ Treatment*Time + (1|Patient ID).

In this study, metabolomics analyses covering 114 small polar molecules were carried out on plasma from participants with type 2 diabetes (n=102), randomized to receive either liraglutide or placebo treatment for 26 weeks on top of their current treatment (**Table 1**) (21). We found that the free fatty acid palmitoleate was significantly lower in the liraglutide treated group compared to placebo (adj. p-value = 0.04) and in extension, that the enzymatic activity of SCD1 was significantly downregulated after liraglutide treatment compared to placebo (p-value = 0.01) as visualized in **Figure 1**.

**Table 1:** Clinical characteristics of participants in The LiraFlame Study at baseline.

	*Randomized to*	
	Liraglutide	Placebo
*Number*	51	51
*Women (%)*	6 (11.8%)	10 (19.6%)
*Mean age in years (SD)*	65.9 (8.6)	66.9 (7.8)
*BMI in kg/m^2^ (SD)*	30.5 (5.3)	29.3 (3.8)
*HbA_1C_ in mmol/mol (SD)*	58.7 (9.6)	58.0 (10.6)

### Palmitoleate Is Lowered by Liraglutide

Polar metabolites were measured applying untargeted metabolomics and targeted (n=31) molecules were fully quantified using heavy labeled isotopes (19, 23). Initially, the association between the metabolite profile and liraglutide treatment was investigated by mapping metabolites to metabolic pathways. Pathways containing four or more measured metabolites were included for further analysis. Notably, we did not observe any significant change in glycolysis, gluconeogenesis or pyruvate metabolism (**Supplementary Table 2**). The pathway containing four molecules pertaining to SCD1 metabolism showed nominal downregulation in response to liraglutide treatment compared to placebo (p-value = 0.08), which led to further investigation of the metabolites within. Fatty acid changes were investigated using linear mixed models, all showed a lower (1-2%) concentration at the end of the trial compared to the baseline in the group treated with liraglutide, this was not the case for the placebo group (**Table 2**). Palmitoleate showed a significant decrease of 4.2% (adj. p-value = 0.04) following liraglutide treatment compared to placebo (**Table 2**). Adjustments for sex, change in HbA_1C_, treatment dose, lipid lowering medication and thiazolidinedione treatment did not affect this result (**Supplementary Table 3**). Adjustment for change in BMI changed the adjusted p-value from 0.04 to 0.06 (**Supplementary Table 3**). Participants receiving liraglutide showed significant improvements in body weight (21), prompting us to investigate if the effect of liraglutide on palmitoleate was mediated by change in BMI. Spearman correlation showed no strong correlation between level of palmitoleate and BMI (**Supplementary Figure 1**). Mediation analysis resulted in a non-significant casual mediation effect (p=0.09) indicating that change in BMI was not mediating the effect of liraglutide on palmitoleate (**Supplementary Table 4**).

**Table 2:** Change in SCD1 metabolites and association to liraglutide and placebo treatment.

	*Average percentage change (SD)*		*Linear mixed model effect of treatment*		*Cohens ds (CI)*
	*Liraglutide*	*Placebo*	*Coefficient*	*p-value*	*Effect size*
*Palmitate (C16:0)*	- 1.3% (4.6)	- 0.3% (4.0)	0.05	0.18	- 0.4 (0.0 -0.8)
*Palmitoleate (C16:1 n-7)*	- 4.2% (14.3)	+ 3.1% (15.9)	0.23	0.04*	- 0.5 (-0.1 -0.9)
*Stearate(C18:0)*	- 1.6% (4.6)	- 0.5% (3.9)	0.05	0.18	- 0.2 (0.2 -0.6)
*Oleate (C18:1 n-9)*	- 1.8% (7.7)	+ 0.5% (6.4)	0.12	0.11	- 0.4 (0.0 -0.8)
*SCD1 activity*	+ 14.5% (43.1)	- 4.1% (22.6)	0.01	0.01**	- 0.5 (-0.1 -0.9)

Average percentage change is calculated for each individual as (end of the trial – baseline)/baseline * 100. Then average and standard derivation was calculated. Linear mixed models were constructed for each metabolite (and SCD1 activity) with the following formular: x ~ treatment type*time point + (1|patient ID). Linear mixed models were fitted using the lme4 package in R. All metabolites where adjusted for multiple testing, SCD1 activity was not. Standardized mean difference of two treatment groups were calculated using the effsize package in R. *Signifies a p-value <0.5; ** signifies a p-value <0.01.

### SCD1 Activity Is Decreased by Liraglutide

Since lower levels of palmitoleate could be indicative of dampened SCD1 activity, we focused on understanding the association between SCD1 activity and liraglutide treatment. SCD1 is the rate limiting enzyme for converting saturated fatty acids (SFAs) into monounsaturated fatty acids (MUFAs), specifically the conversion of palmitate (also called palmitic acid or C16:0) into palmitoleate (palmitoleic acid or C16:1n7) and stearate (stearic acid or C18:0) into oleate (oleic acid or C18:1n9). SCD1 activity has been accurately approximated as the ratio between circulating palmitoleate and palmitate (31, 33). SCD1 activity was downregulated by liraglutide treatment compared to placebo (p-value < 0.01) (**Figure 1**), adjustment for sex, change in BMI, HbA_1C_ and lipid lowering medication did not affect this result (**Supplementary Table 3**).

**Figure 2:**
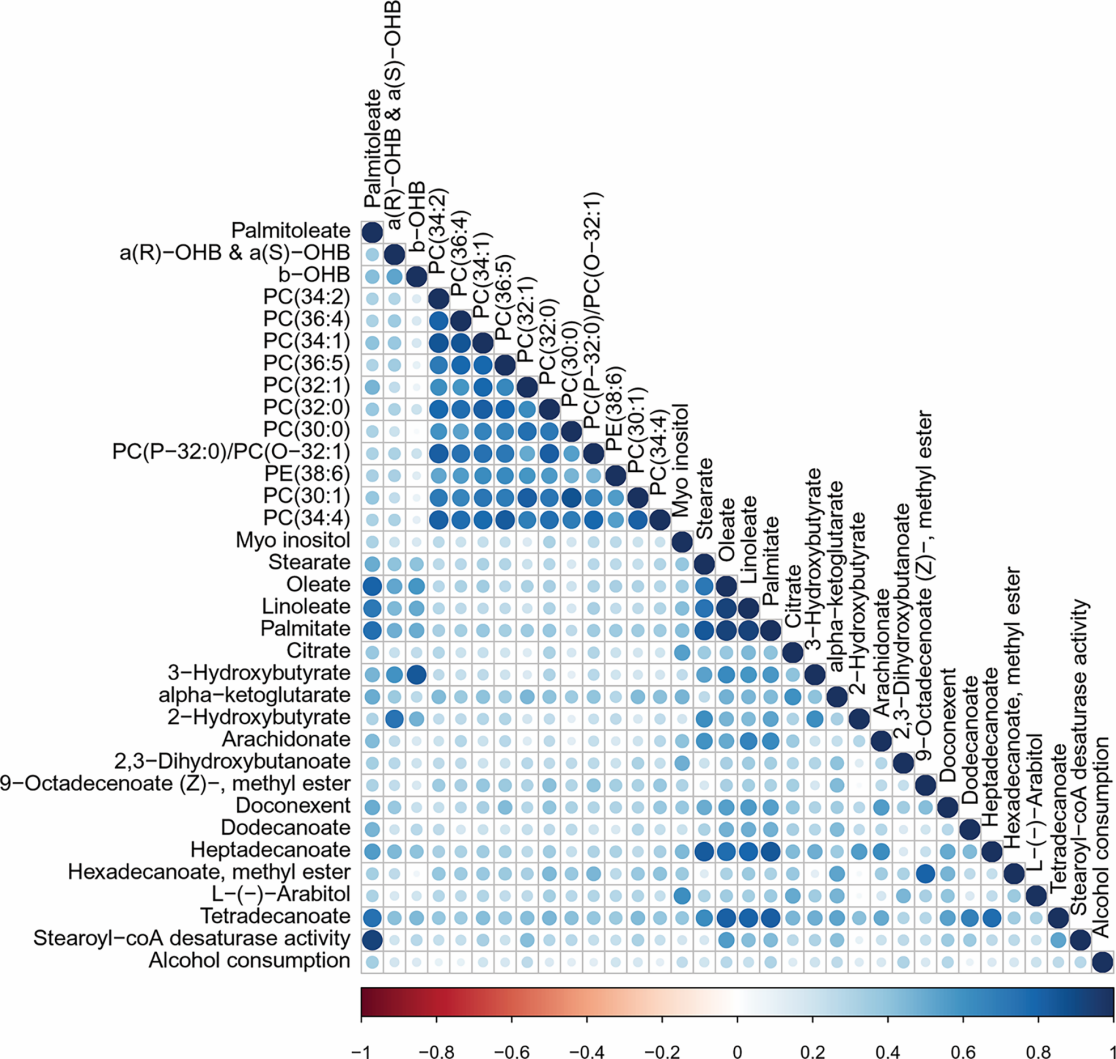
Correlation matrix for palmitoleate. Variables correlated with palmitoleate. Correlation matrix plotting Pearson correlations for variables with more than 30% correlation to palmitoleate. Showing 33 out of 816 variables: 214 clinical measurement, 261 lipids, 117 untargeted metabolites, 193 unannotated metabolites, 31 targeted metabolites. Visualized using the corrplot package in R.

Correlation matrices were computed to further explore metabolic changes. Lipidomic data from our previous work was added for these additional multi-layer analyses (18). Palmitoleate correlated with other free fatty acids such as palmitate, oleate, and tetradecanoate (myristic acid), to several phosphtatidylcholines and to alcohol intake (**Figure 2**). The SCD1 activity associated to fatty acids and phosphtatidylcholines similar to palmitoleate (**Supplementary Figure 2**).

## Discussion

In the present study we found that palmitoleate showed a significant decrease with liraglutide treatment. However, there is conflicting evidence when reviewing the bioactivity of palmitoleate in the literature. Several clinical studies found that increased palmitoleate was associated with increased insulin sensitivity (36–38), suggesting for it to be a lipokine with protective metabolic properties (39). On the other hand, have other clinical studies reported that higher levels of palmitoleate were associated with increased insulin resistance (40–42). The discrepancies could be ascribed to differences in cohorts with varying health status and BMI, being the reason why in our study we analysed the data by adjusting for several variables including BMI (42, 43). We observed that the levels of circulating palmitoleate associated to alcohol intake in concordance with previous observations (41, 44).

Here we showed that the SDC1 activity was significantly reduced in the liraglutide group compared to placebo. Interestingly, an equivalent activity reduction to the levels found here, was achieved by Corpeleijn et al. in a lifestyle intervention of diet and exercise which improved insulin sensitivity and reduced SCD1 activity as measured in serum (30). Studies performed in rodent models have also shown that inhibition of SCD1 improved insulin sensitivity and prevented diet-induced obesity (45–47). While the opposite direction of change, increased level of palmitoleate and SCD1 activity, has been associated with a higher risk of heart failure (32) and mortality (48).

SCD1 is regulated by multitude of factors and the two major influencers are insulin and leptin (49, 50). SCD1 is activated by insulin (51, 52) and inhibited by leptin (53–55). Iepsen et al. found that liraglutide caused leptin to be retained in circulation for longer (56), which might help explain the inhibition of SCD1 we observe. Other studies found the amount of leptin decrease after liraglutide treatment (17, 57). It is possible that glucagon (and thus glucagon mimicking compounds) can affect SCD1 directly as one study showed impaired SCD1 gene expression following glucagon treatment in chicken hepatocytes (58). Statins and thiazolidinediones have been observed to lower SCD1 expression in cell cultures (59, 60), adjustments for statins (lipid lowering medications) and thiazolidinediones did not affect the results presented here (**Supplementary Table 3**).

It is worth noting that the reduction in palmitoleate and SCD1 activity was observed without accumulations of the precursor, palmitate, in circulation. Palmitate is known to induce apoptosis in beta-cells and endothelial cells leading to insulin resistance and atherosclerosis, respectively (61–63). Liraglutide has been shown to protect against the lipotoxicity induced by palmitate in both beta-cells and endothelial cells, this have been suggested to be part of the cardio-protective attributes of liraglutide (64, 65). Here we show that the ratio of palmitoleate to palmitate is decreased by liraglutide treatment in a clinical trial, however the decrease in palmitate was not significantly different between the treatment and placebo group.

It is expected that regulation of SCD1 activity could affect the entire lipidome (49, 66). For instance, palmitoleate and oleate are important substrates for the biosynthesis of larger lipids, especially triglycerides, phospholipids and cholesterol esters (49). Dobrzyn et al, 2005 found that SCD1 deficiency reduced the amount of ceramides by around 40% (67). We previously reported decreases in phosphtatidylcholines, triglycerides and ceramides after liraglutide treatment compared to placebo in this cohort (18), the decrease was stronger in highly unsaturated lipids, suggesting that a reduction in SCD1 activity and availability of MUFAs is involved.

To our knowledge this is the first time liraglutide has been shown to impact MUFA dynamics in humans. The downregulation of palmitoleate and SCD1 activity observed could help explain a favorable cardiovascular profile observed with GLP-1 RA treatment. A major strength of our findings is that metabolomics was carried out in a well characterized double-blinded randomized clinical trial. The LiraFlame Study showed improvement in HbA_1C_ and body weight, but reduced vascular inflammation was not observed (21). Technically experiments were performed in two metabolomics platforms and molecules measured in both showed high correlation, for example, glutamic acid corr = 0.90 (0.87-0.93, p-value < 2.2e-16) due to accurate analytical pipelines.

Here we report that Liragutide reduces the levels of palmitoleate and SCD1 activity suggesting that this mechanism could explain in part downstream metabolic changes beyond the incretin system such as improving lipid profile and reducing the risk of cardiovascular complications.

## Data Availability Statement

The dataset analyzed here is not publicly available, for the privacy of the participants, in compliance with EU and Danish data protection law. The data can be accessed upon request; relevant legal permission from the data protection agency is required. Data access request should be directed to PR, peter.rossing@regionh.dk.

## Ethics Statement

The studies involving human participants were reviewed and approved by the regional ethics committee for RegionH (H-16044546) and the Danish Medicines Agency (2016110109) and was performed in compliance with the principles of the Declaration of Helsinki. The patients/participants provided their written informed consent to participate in this study.

## Author Contributions

AW, EZ, AZ, RR, BS, TH, AK, HV, PR, and CL-Q contributed to the conceptualization and interpretation of this study. EZ, VR, and TH conducted the original clinical trial and provided material and clinical data for this study. AW and IM performed the mass spectroscopy analysis. AW, IM, and AZ did data curation. AW performed the data analysis and drafted the manuscript. All authors approved the final version of the manuscript. AW is responsible for the integrity of the work as a whole.

## Funding

The study was funded by Novo Nordisk and Skibsreder Per Henriksen, R. og hustrus fund. Steno Diabetes Center Copenhagen and Department of Clinical Physiology, Nuclear Medicine & PET, Rigshospitalet have provided internal funding.

## Conflict of Interest

EZ and BS are now employees of Novo Nordisk A/S, work related to this article was done when EZ was employed by Steno Diabetes Center Copenhagen. TH, RR, and PR have shares in Novo Nordisk A/S.

The remaining authors declare that the research was conducted in the absence of any commercial or financial relationships that could be construed as a potential conflict of interest.

## Publisher’s Note

All claims expressed in this article are solely those of the authors and do not necessarily represent those of their affiliated organizations, or those of the publisher, the editors and the reviewers. Any product that may be evaluated in this article, or claim that may be made by its manufacturer, is not guaranteed or endorsed by the publisher.
