# Supplementary material for: Liraglutide Lowers Palmitoleate Levels in Type 2 Diabetes. A Post Hoc Analysis of the LIRAFLAME Randomized Placebo-Controlled Trial
Source: Front Clin Diabetes Healthc. 2022 Mar 4;3:856485. doi: 10.3389/fcdhc.2022.856485 (PMC10012104; doi:10.3389/fcdhc.2022.856485)
Supplement: Supplementary file 1 [file DataSheet_1.pdf]

Supplementary Material:

Supplementary Table 1.

Clinical characteristics based on dosage groups

	Per protocol participants	*Participants with adjusted protocol that reached a dose of 1.8 mg/day*	*Participants ending on a lower tolerable dose than 1.8 mg/day*	*Participants that discontinued treatment*
Number	70	8	12	12
Liraglutide (%)	28 (40%)	4 (50%)	10 (83.3%)	9 (75%)
Mean number of days to max tolerable dose (SD)	18.6 (10.4)	25.1 (29.5)	39.2 (42.8)	NaN
Women (%)	9 (12.9%)	1 (12.5%)	3 (25%)	3 (25%)
Mean age in years (SD)	66.2 (8.0)	62.3 (7.6)	68.5 (7.2)	68.1 (10.0)
Mean BMI in kg/m2 (SD)	30.4 (4.9)	29.7 (3.1)	27.6 (3.8)	29.4 (4.3)
Mean HbA1C in mmol/mol (SD)	57.6 (9.3)	64.5 (17.4)	59.5 (8.4)	57.7 (9.4)

Supplementary Table 2

Pathway	*LMM coefficient*	*LMM P-value*
Glycolysis, Gluconeogenesis, and Pyruvate Metabolism	-0.02	0.90
Leucine, Isoleucine and Valine Metabolism	0.04	0.85
*SCD*1 *Metabolism*	0.39	0.08
Primary Bile Acid Metabolism	0.17	0.15
Secondary Bile Acid Metabolism	0.14	0.12
TCA Cycle	0.01	0.94
Tryptophan Metabolism	-0.03	0.84
Urea cycle; Arginine and Proline Metabolism	-0.08	0.52

Linear mixed models were constructed for each pathway with the following formula: x ~ treatment type*time point + (1|patient ID). Linear mixed models were fitted using the lme4 package in R.

Supplementary Table 3

Linear mixed model of association to liraglutide treatment compared to placebo sensitivity analysis						
Estimate (p-value)						
	Unadjusted	*Adjusted for change in BMI*	*Adjusted for lipid lowering medication and thiazolidinedione treatment*	*Adjusted for treatment dose groups*	*Adjusted for sex, change in BMI, change in HbA1c, lipid lowering medication and thiazolidinedione treatment*	*Adjusted for sex, change in HbA1c, lipid lowering medication and thiazolidinedione treatment*
Palmitate (C16:0)	0.05 (0.18)	0.05 (0.20)	0.05 (0.19)	0.05 (0.17)	0.05 (0.21)	0.05 (0.20)
Palmitoleate (C16:1 n-7)	0.23 (0.04*)	0.22 (0.06)	0.22 (0.04*)	0.22 (0.04*)	0.22 (0.06)	0.23 (0.04*)
Stearate (C18:0)	0.05 (0.18)	0.04 (0.20)	0.04 (0.19)	0.05 (0.17)	0.04 (0.21)	0.04 (0.20)
Oleate (C18:1 n-9)	0.12 (0.11)	0.11 (0.14)	0.11 (0.12)	0.11 (0.11)	0.11(0.14)	0.11 (0.12)
SCD1 activity	0.17 (0.01**)	0.17 (0.01**)	0.17 (0.01**)	0.17 (0.01**)	0.17 (0.01**)	0.17 (0.01**)

Linear mixed models were constructed for each metabolite (and SCD1 activity) with the following formula: x ~ treatment type*time point + adjustment(s) + (1|patient ID). Linear mixed models were fitted using the lme4 package in R. All metabolites where adjusted for multiple testing, SCD1 activity was not. Dose groups were defined as 1: participants following trial protocol, 2: participants that momentarily reduced treatment dose or paused but ended the trial at full treatment dose, 3 participants that ended the trial with a reduced treatment dose (>1.8 mg/day), 4: participants that discontinued treatment. Characteristics of these groups can be found in supplementary table 5

Supplementary Table 4

	Mediation analysis of change in BMI							
	Total Effect Estimate	*Total Effect CI Lower*	*Total Effect CI Upper*	*Total Effect p-value*	*ACME Estimate*	*ACME CI Lower*	*ACME CI Upper*	*ACME p-value*
Palmitate	-0.60	-5.82	4.14	0.43	-0.03	-0.07	0.01	0.22
Palmitoleate	-0.46	-1.50	0.22	0.11	-0.09	-0.20	0.01	0.09
Stearate	-0.23	-7.06	4.15	0.86	-0.01	-0.05	0.03	0.70
Oleate	-0.63	-8.51	6.52	0.20	-0.06	-0.15	0.01	0.10
SCD1	-0.42	-1.25	0.04	0.07	-0.07	-0.17	0.01	0.07

The mediations of change in BMI for the treatment effect on metabolite were investigated by creating linear regression models with and without BMI adjustment, the mediation effect and its significance were then calculated by bootstrapping 500 samples with the mediator package in R. ACME is the average casual mediation effect.

Supplementary Figure 1

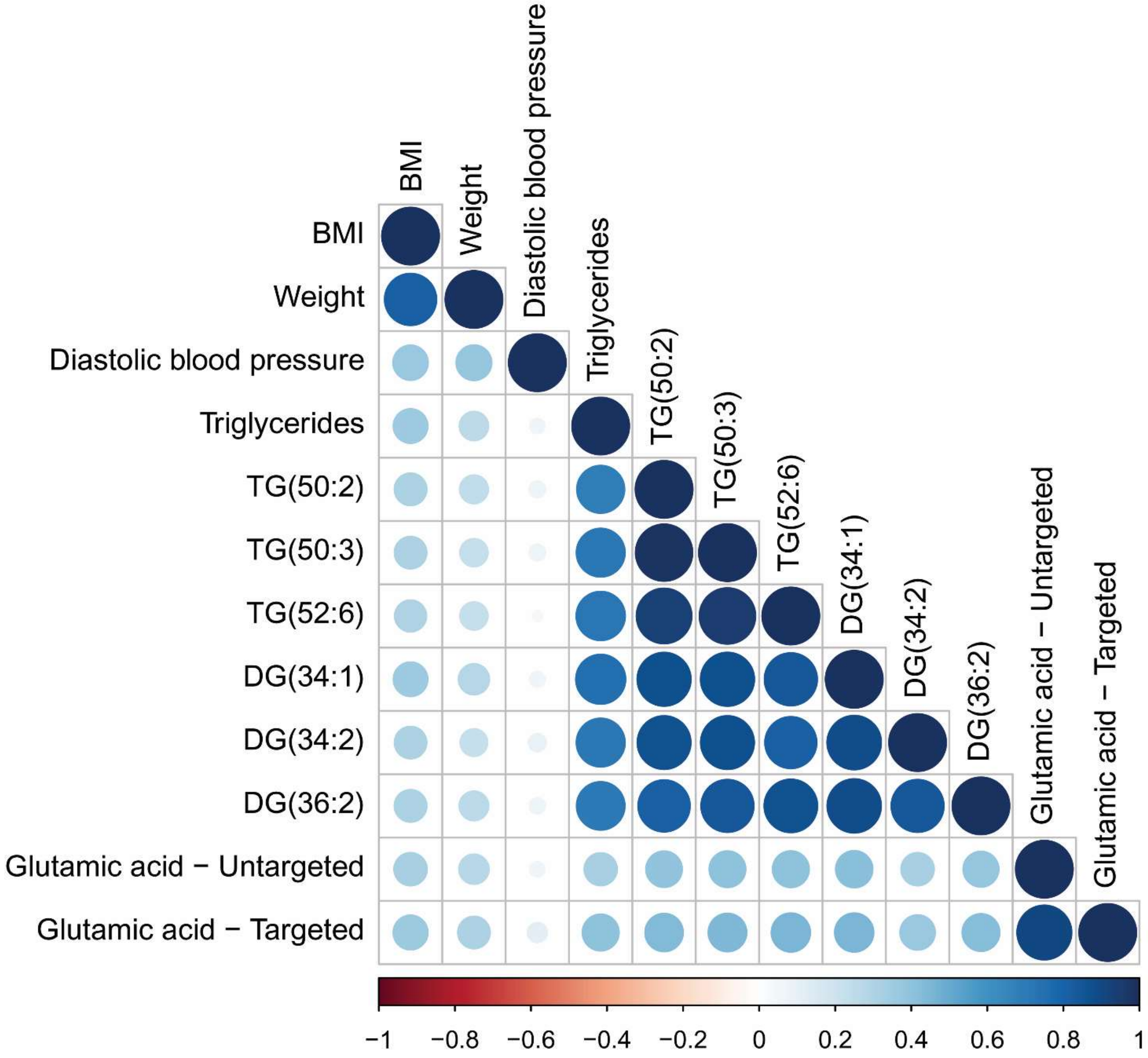

Variables correlated with BMI. Correlation matrix plotting Pearson correlations for variables with more than 30% correlation to BMI. Showing 11 out of 816 variables: 214 clinical measurement, 261 lipids, 117 untargeted metabolites, 193 unannotated metabolites, 31 targeted metabolites. Visualized using the corrplot package in R.

Supplementary Figure 2

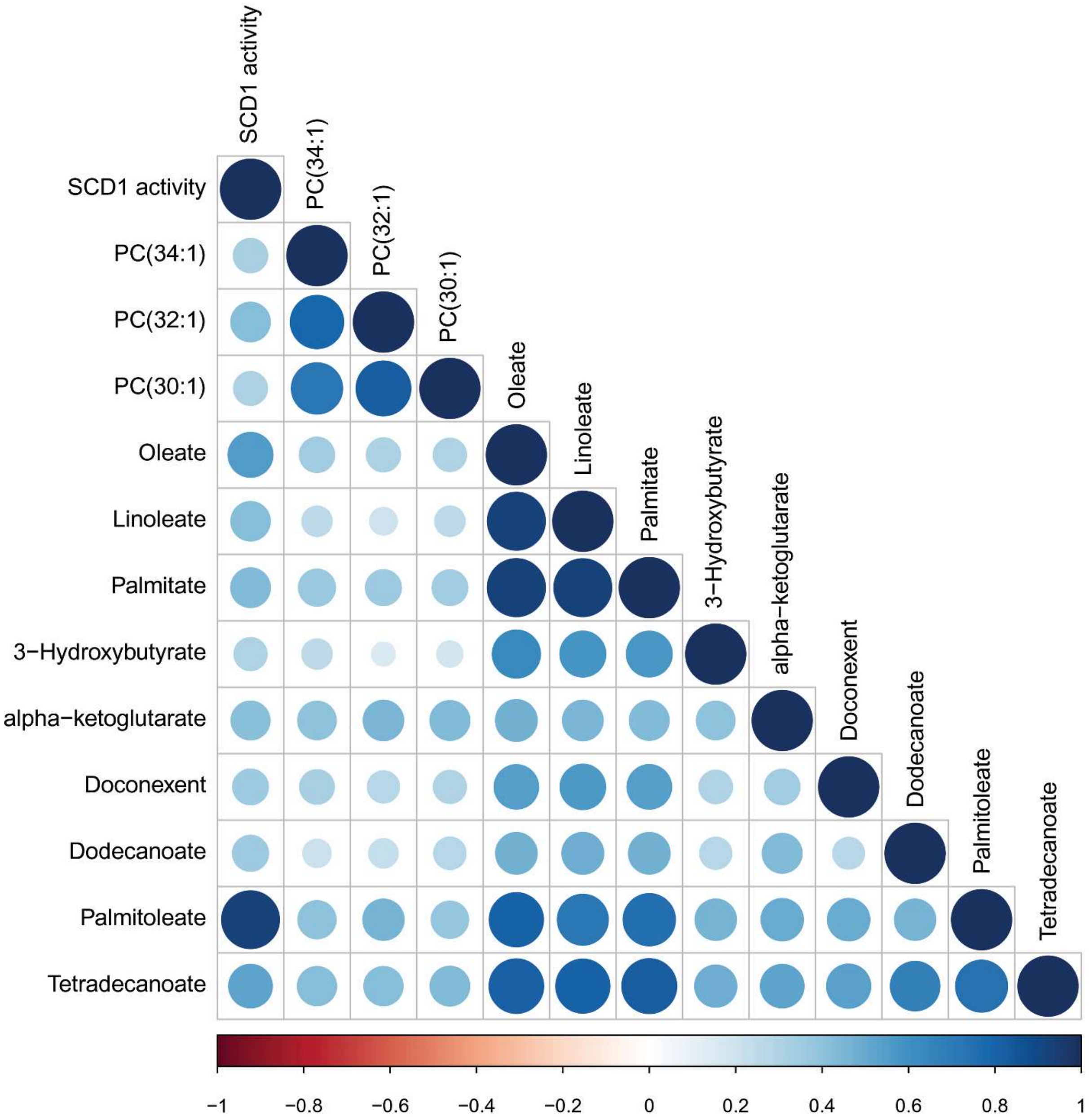

Variables correlated with SCD1 activity. Correlation matrix plotting Pearson correlations for variables with more than 30% correlation to SCD1 activity, estimated by the ratio of palmitoleate/palmitate. Showing 12 out of 816 variables: 214 clinical measurement, 261 lipids, 117 untargeted metabolites, 193 unannotated metabolites, 31 targeted metabolites. Visualized using the corrplot package in R.
